# Administration of small-molecule guanabenz acetate attenuates fatty liver and hyperglycemia associated with obesity

**DOI:** 10.1038/s41598-020-70689-5

**Published:** 2020-08-13

**Authors:** Satoshi Yoshino, Yusaku Iwasaki, Shunichi Matsumoto, Tetsurou Satoh, Atsushi Ozawa, Eijiro Yamada, Satoru Kakizaki, Juan Alejandro Oliva Trejo, Yasuo Uchiyama, Masanobu Yamada, Masatomo Mori

**Affiliations:** 1 Department of Medicine and Molecular Science, Gunma University Graduate School of Medicine, Maebashi, 371-8511 Japan; 2 Laboratory of Animal Science, Graduate School of Life and Environmental Sciences, Kyoto Prefectural University, Kyoto, 606-8522 Japan; 3 Department of Cellular and Molecular Neuropathology, Juntendo University School of Medicine, Tokyo, 113-8421 Japan; 4 Metabolic and Obese Research Institute, Maebashi, 371-0048 Japan

**Keywords:** Drug discovery, Physiology, Endocrinology, Gastroenterology

## Abstract

Nonalcoholic fatty liver disease (NAFLD) is characterized by excessive accumulation of hepatic triglycerides (TG) and hyperglycemia arising due to persistent insulin resistance, and is profoundly linked to obesity. However, there is currently no established treatment for NAFLD in obese human subjects. We previously isolated Helz2, the expression of which was upregulated in human and mouse NAFLD, and its deletion activated the hepatic expression of functional leptin receptor long form (Leprb) and suppressed NAFLD development and body weight (BW) gain in obese mice. A high-throughput assay of small-molecule drugs revealed that guanabenz acetate (Ga), originally used to treat hypertension, possesses a high affinity constant against HELZ2, and its administration activates *LEPRB* expression in HepG2 cells in vitro. The chronic oral administration of Ga shows the selective leptin sensitization in the liver via upregulation of hepatic *Leprb* expression, which affects expression of genes involved in lipogenesis and fatty acid β-oxidation and diminishes hepatocyte hypertrophy with droplets enriched in TG in high-fat diet-induced obese mice. This activity significantly improves insulin resistance to decrease hyperglycemia and hepatocyte and adipocyte weights, resulting in BW reduction without reducing food intake. Regarding drug repositioning, Ga has the potential to effectively treat NAFLD and hyperglycemia in obese patients.

## Introduction

NAFLD commonly develops with obesity and is the hepatic component of metabolic syndrome, and its prevalence is increasing worldwide^1^. NAFLD encompasses a wide spectrum of chronic pathological liver conditions ranging from simple hepatosteatosis to NASH and cirrhosis, and occurs in approximately 25% of adults globally^2^. The prevalence of NAFLD is more than 50% among patients with T2DM^3^, and these patients are at an increased risk of liver-related mortality and cardiovascular disease^2, 3^.

The liver consists of various cell types with hepatocytes being the main parenchymal cells, in which lipid droplets, mainly TG, accumulate, and an excessive TG content in hepatocytes is a hallmark of NAFLD^4,5^. An increased liver TG content is a predictor of hepatic insulin resistance, which causes hyperglycemia, independently of visceral adipocyte volumes or body mass index in individuals with obesity^4,5^. Insulin resistance is associated with lipid deposition in the peripheral metabolic organs such as the liver, WAT, BAT and skeletal muscle. Moreover, hepatic steatosis in almost all cases has been shown to precede the development of insulin resistance in other peripheral metabolic organs^4–6^. Decreasing excessive lipid levels in the liver was reported to attenuate insulin resistance, thereby ameliorating hyperglycemia in subjects with T2DM and/or NAFLD^7–9^. A prolonged high blood level of insulin, attributed to hepatic insulin resistance, contributes in the progression of NAFLD to liver fibrosis, NASH and possibly hepatocellular carcinoma^1,10,11^. Effective pharmacological treatments are urgently needed to treat NAFLD in obese patients^2,12^.

Obesity induces not only resistance to the biological effects of insulin but also resistance to those of the adipocyte-derived leptin^13^. Leptin regulates energy expenditure and metabolic actions in peripheral tissues and also acts in the central hypothalamus to suppress food intake^13^. The effects of leptin are dependent on the presence of functional Leprb, which is localized in the brain and peripheral tissues^14,15^. Leprb is expressed in the liver, where leptin, in conjunction with insulin, plays important roles in suppressing TG accumulation and gluconeogenesis^15,16^. However, circulation levels of leptin are elevated in obese individuals, indicating leptin resistance^17^. In addition, previous studies have proposed the concept of selective leptin resistance, whereby not all leptin actions are equally affected^13,18^. Thus, the significant upregulation of *Leprb* expression in the liver might prevent the selective leptin resistance associated with hepatosteatosis in obese patients.

Metabolic homeostasis is mainly regulated at the transcriptional level, and some coregulators act as metabolic sensors and transcriptional effecters in interactions with nuclear receptor transcription factors and the basal transcriptional machinery^19^. We previously isolated and characterized the coregulator Helz2 (or PDIP1), which binds to the DNA binding domain of PPARγ and activates the ligand-dependent transcription. Among the peripheral metabolic organs, Helz2 was found to be strongly expressed in the liver, in contrast to scarce expression in the brain^20,21^. The hepatic expression of *Helz2* was influenced by feeding behaviors and increased with fatty liver in HFD-induced obese mice, suggesting that Helz2 functions as a metabolic sensor in the liver^21^. We also demonstrated that *HELZ2* expression levels were significantly higher in the livers of obese human subjects with NAFLD than in those without^21^. Reduction in *Helz2* expression in obese mice attenuated hepatosteatosis and hyperglycemia, which prevented BW gain without detectable anorexia. Notably, *Helz2* deficiency significantly upregulated the hepatic expression of *Leprb*, but did not change the hypothalamic *Leprb* expression or basal feeding amounts, and exogenous leptin-induced feeding reduction was not observed^21^, the data suggesting reversal of leptin resistance selectively in the liver. Moreover, we showed that *Helz2* suppressed the activity of the leptin receptor promoter^22^ in conjunction with nuclear transcription factors (in preparation). These findings collectively imply that inhibition of *Helz2* function clearly upregulates the *Leprb* expression in the liver and that hepatic Helz2 is a potential target molecule for the treatment of fatty liver and hyperglycemia.

In the present study, we made an attempt to identify a Helz2-associated small-molecule drug that ameliorates fatty liver and hyperglycemia in HFD-induced obese mice.

## Results

### Identification of a small-molecule drug that possesses a high-affinity constant against HELZ2 and activates ***LEPRB*** expression in vitro

We searched for small-molecule drugs that simultaneously fulfilled the following functions, (1) a high-affinity constant against HELZ2 and (2) an increase in *Leprb* expression in vitro (Suppl. Fig. 1-1, 1-2). Among 1,200 small-molecule drugs, a high-throughput screening assay system^23^ identified 14 small-molecule drugs (see chemical compounds) with high-affinity constants ranging from 10^–9^ to 10^–7^ M. Next, we examined the effects of each of these drugs, at the concentrations ranging between 10^–9^ and 10^–7^ M, on *LEPRB* expression in HepG2 cells. Treatment with small-molecule salbutamol showed an affinity constant of 2.2 × 10^–7^ M, but did not significantly affect *LEPRB* expression. Among the drugs tested, treatment with Ga caused a dose-dependent elevation of *LEPRB* expression.

The molecular weight of Ga is 291.1 and its KD value against HELZ2 is 2.3 × 10^–9^ M. Ga is an α2-adrenergic receptor agonist with a Ki value of 7 × 10^–9^ M^24^ and exerts its effects at the central and peripheral levels to decrease blood pressure^24^. The half-life of Ga is 4.3 h, and its estimated efficacy is 12 h. Approximately 75% of orally administered Ga is absorbed by the gastrointestinal tract, and nearly all of Ga is metabolized in the liver. The other drugs used as anti-hypertension treatment via activation of the α2-adrenergic receptor include clonidine hydrochloride, methyldopa and guanfacine hydrochloride, but these drugs showed the very low affinity constants (over 10^–6^ M) for HELZ2.

### The oral administration of Ga reduces hepatic TG content and hyperglycemia in a dose-dependent manner

Based on the maximum dose of Ga used clinically and its estimated 12 h efficacy, and considering the frequency of oral administration to mice (once daily), a medium Ga dose was established as 0.32 mg/kg BW, with 0.11 mg/kg BW as a low-dose and 0.96 mg/kg BW as a high-dose.

**Figure 1:**
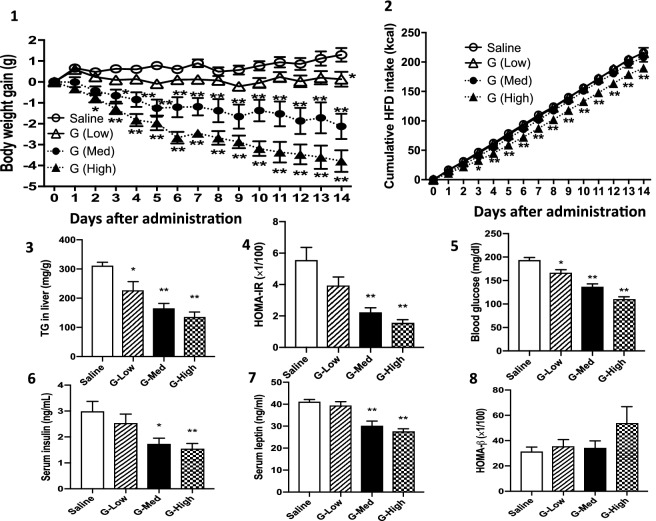
The oral administration of Ga reduces hepatic TG content and hyperglycemia in a dose-dependent manner. BW gain from the initiation day of Ga administration (**1-1**) and cumulative food intake (**1-2**) were measured daily (n = 7 in each group). After 14 days of treatment, hepatic TG content (**1-3**), insulin resistance (**1-4**), blood levels of glucose (**1-5**), insulin (**1-6**) and leptin (**1-7**), and HOMAβ values (**1-8**) were determined. Saline, G-Low, G-Med and G-High represent the saline-treated, low Ga dose-treated, medium Ga dose-treated and high Ga dose-treated groups, respectively. Data are presented as the mean ± SEM. *P < 0.05 and **P < 0.01 compared to the saline-treated group, respectively.

As deletion of *Helz2* protected against HFD-induced obesity in mice with no changes in food intake^21^, we initially examined daily changes in BW and food intake after treatment with various doses of Ga in obese mice. Treatment with Ga for 14 days caused dose-dependent decreases in BW gain (Fig. 1). No significant changes in cumulative food intake were observed in mice administered the low- or medium-dose. In contras, the decrease in food intake was noted after treatment with the high-dose, possibly due to its toxic effects on feeding or its anorexic effects on the brain without involvement of *Helz2* function.

Hepatic insulin resistance and the pancreatic β-cell function of insulin secretion capacity were calculated using HOMA-IR and HOMAβ^25,26^, respectively. The oral administration of Ga caused dose-dependent decreases in metabolic parameters including hepatic TG contents, hyperglycemia with insulin resistance, and blood levels of insulin and leptin. However, HOMAβ values were not significantly affected by any doses of Ga.

All obese mice treated with all doses of Ga were apparently healthy and did not show diarrhea or muscular shivering during all experimental stages.

### The oral administration of the medium-dose of Ga decreases BW gain without reducing food intake in obese mice

**Figure 2:**
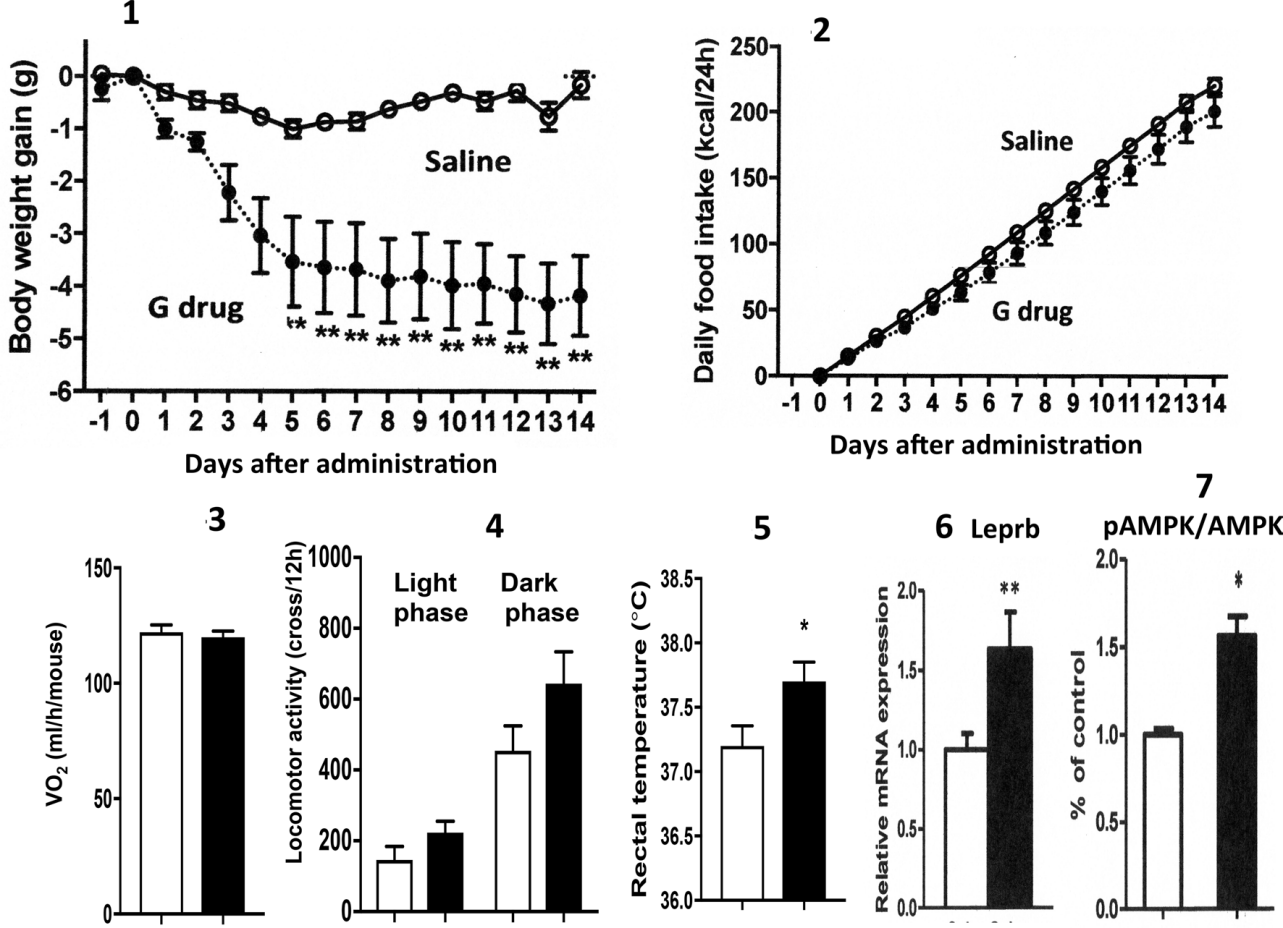
The oral administration of the medium-dose of Ga decreases BW gain without reducing food intake in obese mice. BW gain (**2-1**) and cumulative food intake (**2-2**) were measured (n = 8 in each group). VO2 values (**2-3**), locomotor activities during light and dark phases (**2-4**) and rectal temperature (**2-5**) were estimated (n = 7 in each group). *Leprb* expression (**2-6**, n = 8) and the pAMPK/AMPK ratio (2**-7,** n = 4) in livers were determined. White column and black column represent the saline-treated group and the Ga-treated group, respectively. Data are presented as the mean ± SEM. *P < 0.05 and **P < 0.01 compared to the saline-treated group, respectively.

To investigate the mechanisms, by which Ga attenuated metabolic abnormalities (Fig. 2, Suppl. Fig. S1), the medium-dose of Ga was used, because *Helz2* deficiency attenuated hepatosteatosis and decreased BW gain in obesity. The daily oral administration of the medium Ga dose for 14 days in obese mice resulted in significantly less BW gain, without reducing food intake. The administration of Ga did not increase the basal energy expenditure or locomotor activities, but stimulated core body temperature. Its administration significantly upregulated the hepatic expression of *Leprb*, which stimulated the Leprb-downstream signaling pAMPK.

### The medium-dose of Ga improves hepatocyte hypertrophy in obese mice

**Figure 3:**
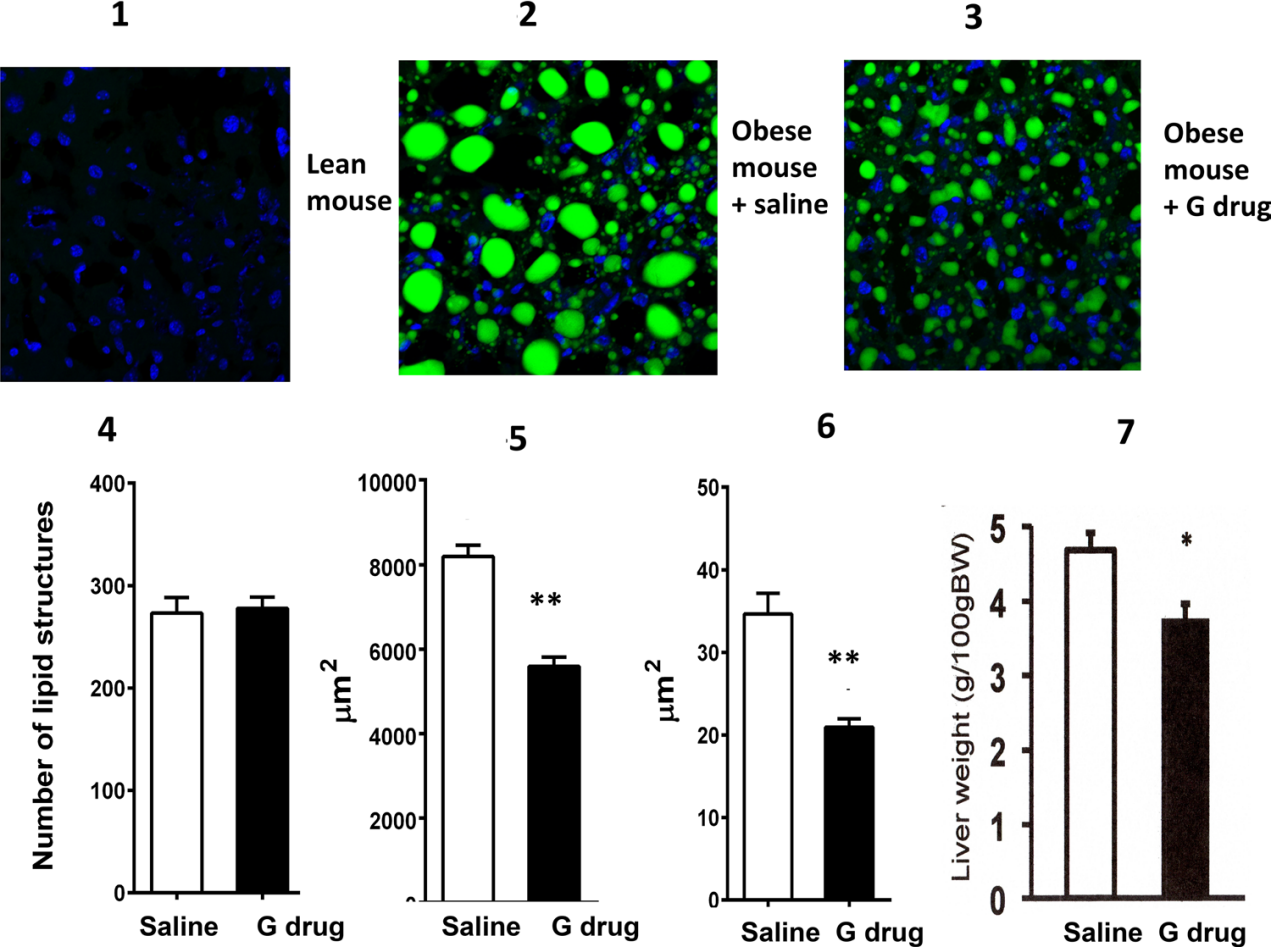
The administration of the medium-dose of Ga improves hepatocyte hypertrophy in obese mice. Hepatic morphologies were examined by means of BODPY staining. The upper panels show representative figures of liver sections stained with BODIPY in LFD-feeding lean mice (**3-1**) and HFD-feeding obese mice treated with saline (**3-2**) or Ga (**3-3**). The degree of hyperplasia (**3-4**) and hypertrophy (**3-5**,**3-6**) in hepatocytes was determined in obese mice (N = 4 in each group). Liver weights relative to BW (**3-7**, n = 8) were evaluated. Data are presented as the mean ± SEM. **P < 0.01 compared to the saline-treated group.

The upper panels show the representative figures of morphological changes in the hepatocytes of obese mice after treatment with Ga (Fig. 3). While the total number of lipid structures in hepatocytes (hyperplasia) remained unchanged, the total area of lipid and average size of lipid structures in hepatocytes (hypertrophy) were significantly reduced by the 14-day administration of Ga. Significant decrease in hepatic volumes was observed after treatment with Ga.

### The medium-dose of Ga affects the hepatic gene expression of lipogenesis and β-oxidation in obese mice

**Figure 4:**
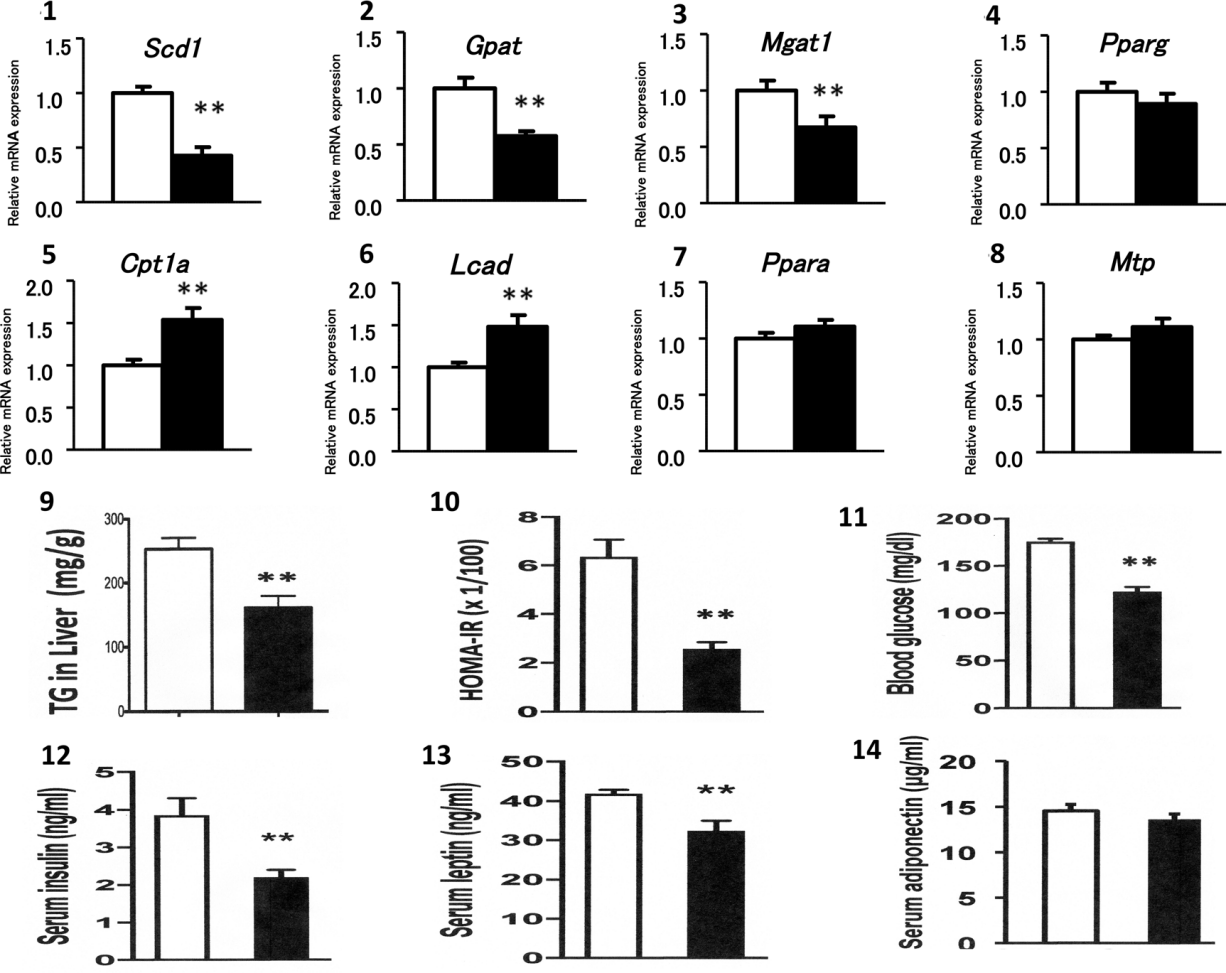
The administration of the medium-dose of Ga affects the hepatic gene expression of lipogenesis and β-oxidation, and the metabolic parameters in obese mice. The expression of genes regulating lipogenesis including *Scd1*, *Gpat*, *Mgat1* and *Pparγ* (**4-1**–**4-4**), and genes regulating β-oxidation or TG export including *Cpt1a*, *Lcad*, *Ppar*α, and *Mtp* expression (**4-5**–**4-8**) was assessed by qRT-PCR (n = 7 in each group). Hepatic TG content (**4-9**), insulin resistance (**4-10**), and blood levels of glucose (**4-11**), insulin (**4-12**), leptin (**4-13**), and adiponectin (**4-14**) were analyzed. White column and black column represent the saline-treated group and the Ga-treated group, respectively. Data are presented as the mean ± SEM. **P < 0.01 compared to the saline-treated group.

As shown in Fig. 4, among the genes regulating hepatic lipogenesis, the administration of Ga significantly downregulated *Scd1*, *Gpat* and *Mgat1* expression, but did not affect *Pparγ, Srebp1c* or *Chrebp* expression (data not shown for the last two genes). Regarding genes that are involved in hepatic FA β-oxidation, the administration of Ga significantly upregulated *Cpt1a* and *Lcad* expression, but did not affect *Ppar*α, *Pparδ*, *Ucp2, Ucp3* or *Pgc1*α expression (data not shown for the last four genes). The hepatic expression of *Mtp* was not altered.

### The medium-dose of Ga decreases hepatic TG contents and hyperglycemia in obese mice

Treatment with Ga reproducibly decreased hepatic TG contents and attenuated insulin resistance, causing significant reductions in blood levels of glucose, insulin and leptin (Fig. 4). Blood levels of adiponectin remained unchanged.

### The medium-dose of Ga affects the adipose insulin resistance index and tissue weights in obese mice

Adipose insulin resistance was assessed using the index of Adipo-IR^27^. Treatment with Ga reduced adipose insulin resistance, and visceral and subcutaneous WAT weights, but not BAT, muscle, or heart weights (Suppl. Fig. 2). Its administration did not affect expression of *Ucp1* in BAT, or expression of *Ucp2* or *Ucp3* in WAT and muscles (data not shown). The blood levels of lipids, such as FFA, CM and VLDL were not altered by treatment with Ga (Suppl. Fig. 3).

## Discussion

The present findings clearly demonstrate that the chronic oral administration of small-molecule Ga, which possesses a high-affinity constant against HELZ2 and activates hepatic *Leprb* expression in vitro and in vivo, prevents the excessive accumulation of TG in hepatocytes and attenuates hyperglycemia associated with insulin resistance in obese mice fed HFD. High blood levels of insulin and leptin were reduced by the Ga treatment. While alterations in taste-related food reward behaviors contribute to BW maintenance^28^, some satiety molecules such as GLP-1 decreased food intake partly through the central effect on taste preference^29^. Mice prefer HFD rather than LFD resulting in obesity, but the intake amounts of HFD were not decreased by the administration of the medium- or low-dose of Ga. In spire of these feeding behaviors, treatment with Ga significantly reduced BW gain in obese mice. Subsequently, we evaluated energy expenditure that consists of three factors^30^; the first of which is resting energy expenditure. The calculation maneuver to determine alterations in energy expenditure remains ill-defined^31,32^, and no significant changes in energy expenditure shown as VO_**2**_ (ml/h/mouse) occurred in mice treated with Ga. The second factor is physical activity monitored as locomotor activities, which showed no changes in mice treated with Ga. The third factor is adaptive thermogenesis shown as core body temperature at the ambient room temperature, and is essentially regulated by the effectors including different types of UCP. In mice treated with Ga, body temperature was slightly but significantly elevated. However, Ga treatment did not activate expression of *Ucp1* in BAT, or expression of *Ucp2* or *Ucp3* in WAT, muscles and livers. Additional effectors have been observed to regulate thermogenic heat production^33,34^, but the findings about whether these effectors contributed to Ga-induced thermogenesis remain undisclosed. The medium-dose of Ga is nearly comparable to that used clinically, and is 3–12.5-fold lower than the chronic treatment doses (1–4 mg/kg BW) employed by previous studies using obese rodents^35,36^. A previous study showed that an intraperitoneal (ip) injection of Ga every other day for 1 week at a dose of 4 mg/kg BW decreased blood insulin levels with increased blood glucose levels, possibly due to its toxic induction of anorexia in HFD-induced obese mice^35^. In contrast, the oral administration of Ga for 3 days at a dose of 1 mg/kg BW increased blood insulin levels with decreased blood glucose levels, and elevated leptin levels with reduced food intake in ob/ob obese mice injected with a leptin-expression vector^36^. The present study is the first to show the chronic in vivo effects of Ga on hepatic excessive TG contents and hyperglycemia due to insulin resistance in obesity.

As leptin physiology is well preserved from mice to humans^37^, much attention has been given to identify certain molecules that enhance the central leptin-sensing systems to reduce appetite and BW in obese mice^38^; for example, small-molecule celastrol acts as a leptin-sensitizer in the brain, leading to apparent reduction in food intake and BW in obese mice^38^. However, many approaches have not been successful in obese humans^37^. In the liver of obese mice, the *Leprb* expression has been observed to be suppressed^39^, and treatment with exogenous leptin did not affect hepatic metabolic functions in obese mice^40^, indicating the concept of leptin resistance in the liver, as well as the central brain^13,18^. The present findings provide a new insight into evidence that small-molecule Ca acts as a selective leptin-sensitizer in the liver through upregulation of endogenous *Leprb* expression, which attenuated hepatosteatosis, leading to improvement of insulin resistance and hyperglycemia in obese mice. In line of this view, the hepatic overexpression of exogenous *Leprb* has been reported to attenuate hepatic TG contents in *Leprb*-deficient *fa/fa* obese rodents^41^. Using the adenovirus-mediated *Leprb* expression vectors, we previously showed that the liver-specific overexpression of *Leprb* in HFD-induced obese mice ameliorated metabolic abnormalities and protected against BW gain, without reducing food intake^21^. Although the administration of exogenous leptin reportedly accelerated the development of NASH in a NASH rodent model, in which the hepatic *Leprb* expression remained detectable^42^, clinical findings have revealed that treatment with exogenous leptin attenuated hepatosteatosis and hepatocellular ballooning injuries seen in NASH patients with leptin-deficient lipodystrophy. These results suggest that the endogenous leptin signaling possibly possesses the ability to reverse abnormalities seen in NASH^43^.

Dysfunctional adipocytes with increased TG storage leading to insulin resistance are characterized by hypertrophy rather than hyperplasia^44^. Hypertrophy and hyperplasia are also observed in hepatocytes with excess lipid contents in obese mice. Treatment with Ga significantly reduced hypertrophy, but not hyperplasia, leading to decreased hepatocyte volumes. Lipid levels in hepatocytes are regulated by the interplay between the delivery of FFA to the liver, hepatic de novo lipogenesis, FA oxidation, and TG export turnover^2,5^. Hepatosteatosis is caused by alterations in the equilibrium of one or more of these cellular processes. In patients with NAFLD, a stable isotope study demonstrated that the elevated hepatic lipid content was largely attributed to de novo lipogenesis^45^. In the present study, treatment with Ga significantly reduced the hepatic expression of lipogenesis-related genes such as *Scd1, Gpat* and *Mgat1* in obese mice. SCD1 has been recognized as a molecule that is targeted and downregulated by leptin in the liver^46^, and SCD1 is the rate-limiting enzyme for hepatic de novo lipogenesis. The liver-specific deletion of *Scd1* in obese mice decreased hepatic lipogenesis, but unexpectedly caused hyperglycemia^47^. Moreover, treatment with a SCD1-specific antagonist in patients with NAFLD decreased hepatic lipogenesis, but did not improve hyperglycemia or insulin resistance^48^. The greater intrahepatic fat storage did not always correlate to pathological insulin resistance and hyperglycemia particularly in certain cases combined with higher oxidative capacity or increased turnover rates^49^. These findings have collectively raised the idea that successful reduction in the metabolic abnormalities due to hepatosteatosis requires activation of FA β-oxidation and/or an increased turnover rate^49^, in addition to reduction of lipogenesis. The Ga treatment not only diminished hepatic *Scd1* expression, but also stimulated the leptin-downstream signal pAMPK to upregulate hepatic *Cpt1a* and *Lcad*, both involved in mitochondrial FA β-oxidation. Ga treatment did not affect hepatic *Mtp* expression, which regulates TG export as VLDL from the liver, without affecting blood VLDL levels. Moreover, SCD inhibition reportedly suppressed the proliferation of hepatocellular carcinoma^50^.

PPARγ is highly expressed in WAT. The hepatic expression of *Pparγ* was elevated in HFD-induced obese mice. The administration of an activator of *Pparγ* accelerated hepatosteatosis^51^, which was abolished by the liver-specific deletion of *Pparγ*^52^. However, treatment with small-molecule pioglitazone, a PPARγ agonist, attenuated insulin resistance in patients with NAFLD and NASH^53^, but increased WAT and BW volumes. In the present study, reduction in adipose and body weights occurred after treatment with Ga. Interestingly, intrahepatic lipid accumulation activated inflammatory immune cells including innate Kupffer cells and bone marrow-derived macrophages that secrete inflammatory mediators^54,55^, causing chronic low-grade inflammation in the liver and obesity-induced insulin resistance^55–57^. NAFLD is also associated with alterations in the composition of gut microbiota that disturb hepatic immune systems^58^. Thus, in the peripheral metabolic organs, the inflammatory cell action is a determinant of maintenance of metabolic disease pathogenesis^57^. Although PPARγ activation in WAT stimulated anti-inflammatory regulatory T cells, which protected against age-related insulin resistance^59^, it remains to be explored whether the administration of Ga may affect the hepatic inflammatory processes to attenuate hepatosteatosis.

Taken all together, the present studies reveled that the chronic oral administration of small-molecule Ga caused the selective leptin sensitization in the liver, resulting in significant attenuation of hepatosteatosis and hyperglycemia in obesity. The concept of drug repositioning^60^ implies the application of an existing therapeutic agent to a new disease indication, which provides a rapid clinical impact at a lower cost than de novo drug development. Accordingly, drug Ga, which is clinically used to treat hypertensive patients, might be clinically applied to treat NAFLD and hyperglycemia in obese patients.

## Materials and methods

All experiments were performed in accordance with relevant guidelines and regulations.

### High throughput assay of small-molecule drugs against HELZ2

FLAG-CMV10 constructs with HELZ2 expression vectors or vacant vectors (control) were transfected into HEK293 cells, and the proteins were purified using anti-FLAG antibody columns. An array panel of 1,200 small-molecule drugs, all approved by the FDA, was used to determine the binding affinity constants based on the association and dissociation of each drug against HELZ2 by means of a high-throughput screening assay system, which was monitored by surface plasmon resonance^23^ (Plexera KKK, Japan).

### Chemical compounds

The following small-molecule drugs showed high-affinity constants against HELZ2, and were purchased from Sigma-Aldrich Co, Japan; amifostine trihydrate, brompheniramine maleatem, clemastine fumarate, fenbendazole, fulvestrant, Ga, loperamide hydrochloride, pirenperone, ritodrine hydrochloride, rivastigmine tartrate, salbutamol, sibutramine hydrochloride, sulbactam, voriconazole, and zoxazolamine.

### In vitro activity assay of drugs

Each of the drugs, at doses of 10^–9^–10^–7^ M, was incubated in HepG2 cells (10^5^ cells per each well in 6-well plates) in DMEM containing 10% fetal bovine serum as described previously^21^. After 48 h, LEPRB gene expression was analyzed by qRT-PCR.

### Animals

All animal protocols were approved by the Institutional Animal Care and Use Committee of Kyoto Prefectural University. Male mice (C57BL6J) were kept at room temperature (23 ± 2 °C) under a 12/12 h light/dark cycle (7:30 light-on and 19:30 light-off). Six-week-old mice were individually housed and fed either HFD (DI2492) with 60 kcal% fat, supplied by Research Diet Inc., or LFD (standard chow, CE-2) with 12.3 kcal% fat, supplied by CLEA Inc., Japan. After feeding for 18 weeks, mice fed the HFD were divided into 4 groups for the dose–response experiment or 2 groups for experiments on the medium-dose. Using a gastric intubation tube, each mouse was orally administered the drug in 0.3 ml saline once daily 60 min before lights off for 14 consecutive days. On the 15th day, food intake was stopped at 9:00, and mice were euthanized by isoflurane inhalation at 14:00. Blood and organ samples were stored at − 80 °C until assayed.

### Assay of blood metabolic parameters

Blood glucose was measured following an enzyme method. Blood insulin and leptin levels were analyzed with a mouse ELISA kit (Morinaga Institute of Biological Science, Inc., Japan), and blood adiponectin levels were measured with a mouse adiponectin ELISA kit (Otsuka Pharmaceutical Co., Ltd.). Lipid levels in the blood and the liver were analyzed by the LipoSearch method using high performance liquid chromatography (Skylight Biotech, Inc. Japan)^21^. Blood FFA levels were measured using the NEFA-c Test Wako kit (Fuji Film Wako Pharmaceutical Co., Japan).

### Evaluation of hepatic insulin resistance and β-cell function

Hepatic insulin resistance was assessed using HOMA-IR as follows^25^; [insulin (mU/L) × blood glucose (mM) ÷ 22.5] index (× 100). Theβ-cell function of insulin secretion capacity was calculated using HOMAβ as follows^25^; [insulin (mU/L) × 20] ÷ [blood glucose (mM) − 3.5] index (× 100). The index of HOMA-IR^25^ is independently associated with a high prevalence of cardiometabolic disorders^26^, and HOMAβ^25^ shows the pancreatic β-cell function of insulin secretion capacity.

### Evaluation of adipose insulin resistance

The method used to assess adipose insulin resistance in the presence of high insulin level was Adipo-IR^27^, which was evaluated as follows; fasting FFA (mmol/L) × insulin (pmol/l). The index of Adipo-IR is a potential method for assessing adipose insulin resistance and is a marker of the ectopic fat deposition^27^.

### qRT-PCR analysis

qRT-PCR was performed as described previously^20,21^. The primers used were the genes encoding Leprb, coregulators, nuclear transcription factors, and genes involved in lipogenesis and FA oxidation. Each gene corresponded to the TacMan gene expression assay as described previously^21^. Amplification was normalized by parallel qRT-PCR of G3PD mRNA.

### Western blot analysis

Western blot analysis was performed as described previously^21^. Whole-cell lysate samples were prepared and proteins were separated by SDS-PAGE, before being transferred to polyviniylidene diffluoride membranes. The transferred membranes were incubated with the Ab specific to AMPKα (AMPK). The AMPK Ab was then removed with a stripping buffer, before membranes were incubated with the Ab to phosphorylated (p)Ther172-AMPKα (pAMPK) . All Abs were purchased from Cell Signaling, Inc. Membranes were incubated with horseradish peroxidase-conjugated secondary Ab (GE Healthcare), and signal was visualized using a Chemilmi image analyzer (Lumino Graph1, ATTO Inc.).

### Histology analysis

Tissue sections were prepared, and immunohistochemistry was performed using the intracellular BODIPY lipid staining method. Hepatic hyperplasia was evaluated by estimating the total number of lipid structures. The degree of hepatic hypertrophy was evaluated by the average of the total area of lipids and the average size of lipid structures. Lipid areas and structures were calculated in 100 cells of each liver section stained with BODIPY in 5-field sections per group.

### Tissue weights

The weights of visceral (mesenteric and perirenal) WAT, subcutaneous WAT, BAT, muscle (right-sided gastrocnemius and soleus muscles), and heart were measured in each obese mouse and shown as weight relative to BW.

### Measurement of oxygen consumption (VO_2_), locomotor activity and body temperature

VO_**2**_ was measured using an indirect calorimetry system (Oxymax, Colombus Instrument OH) as described previosuly^21^. HFD-induced obese mice were individually placed in the small acryl calorimeter chamber with free access to HFD and water from experiment day 12 to the end of experiment. After 12 h of adaptation, VO_**2**_ was estimated in individual mouse for 1 min at a 15-min interval over a 24 h period at airflow of 0.71/min. VO_**2**_ values were shown as ml/h/mouse. Locomotor activity of each mouse was monitored as numbers of infrared beam broken in both X and Y directions using an activity monitoring system (ATIMO-100 N, Shinfactory, Fukuoka, Japan). Rectal body temperature was measured using a thermometer (BAT-12R and RET-3, Physitemp).

### Statistical analysis

Data from more than three groups were analyzed by ANOVA followed by Dunnett’s test, and the significance of differences between two groups was assessed by a paired t-test. Data are presented as the mean ± SEM. *P < 0.05 and **P < 0.01 compared to the saline-treated group, respectively.

## Supplementary information

Supplementary Legends

Supplementary Figures

Supplementary Table
