# Supplementary material for: Administration of small-molecule guanabenz acetate attenuates fatty liver and hyperglycemia associated with obesity
Source: Sci Rep. 2020 Aug 13;10:13671. doi: 10.1038/s41598-020-70689-5 (PMC7426972; doi:10.1038/s41598-020-70689-5)
Supplement: Supplementary file 1 — Supplementary Legends [file 41598_2020_70689_MOESM1_ESM.docx]

**Suppl**. **Table** 1. Abbreviations are shown

.

**Suppl. Fig. 1.** **Small-molecule Ga stimulates *LEPRB* expression in cultured cells and activates pAMPK formation in livers.**

LEPRB expression in HepG2 cells incubated with various doses of salbutamol (Suppl. Fig. 1-1, n=3 in each column) or Ga (Suppl. Fig. 1-2, n=6) was assessed by qRT-PCR. Western blots (Suppl. Fig. 1-3) were performed to detect AMPK (the lower panel) and pAMPK (the upper panel). The arrow shows the position of AMPK. S and G indicate the saline- and Ga-treated groups, respectively. Data are shown as the mean ± SEM. *P<0.05 to the vehicle-treated group.

**Suppl. Fig. 2.** **Treatment with the medium-dose of Ga affects the adipose insulin resistance index and tissue weights relative to BW in obese mice.**

The adipo-IR (Suppl. Fig. 2-1) index was determined. The tissue weights of vWAT (Suppl. Fig. 2-2), sWAT (Suppl. Fig. 2-3), BAT (Suppl. Fig. 2-4), muscle (Suppl. Fig. 2-5) and heart (Suppl. Fig. 2-6) were determined (n=7 in each group). Data are presented as the mean ± SEM. *P<0.05 and **P<0.01 compared to the saline-treated group, respectively.

**Suppl. Fig. 3. Treatment with the medium-dose of Ga does not change the blood lipid profile**.

After 14 days of treatment, the blood levels of lipids, including FFA, CM and VLDL (Suppl. Figs. 3-1〜3-3) were assessed. Data are presented as the mean ± SEM (n=7 in each group).
