# Supplementary material for: Administration of small-molecule guanabenz acetate attenuates fatty liver and hyperglycemia associated with obesity
Source: Sci Rep. 2020 Aug 13;10:13671. doi: 10.1038/s41598-020-70689-5 (PMC7426972; doi:10.1038/s41598-020-70689-5)

Administration of small-molecule guanabenz acetate attenuates fatty liver and hyperglycemia associated with obesity

Satoshi Yoshino, Yusaku Iwasaki,
Shunichi Matsumoto, Tetsurou Satoh,
Atsushi Ozawa, Eijiro Yamada, Satoru Kakizaki,
Juan Alejandro Oliva Trejo, Yasuo Uchiyama,
Masanobu Yamada and Masatomo Mori

Suppl. Fig. 1-1

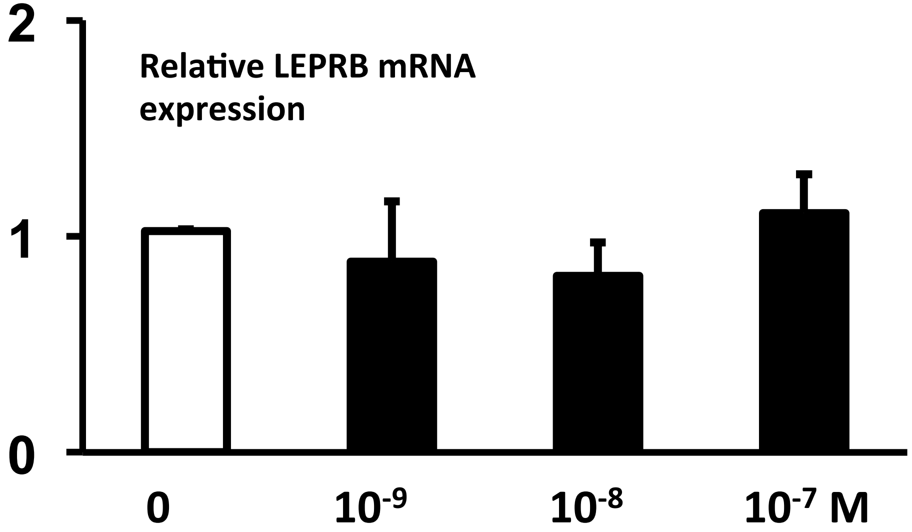

Suppl. Fig. 1-2

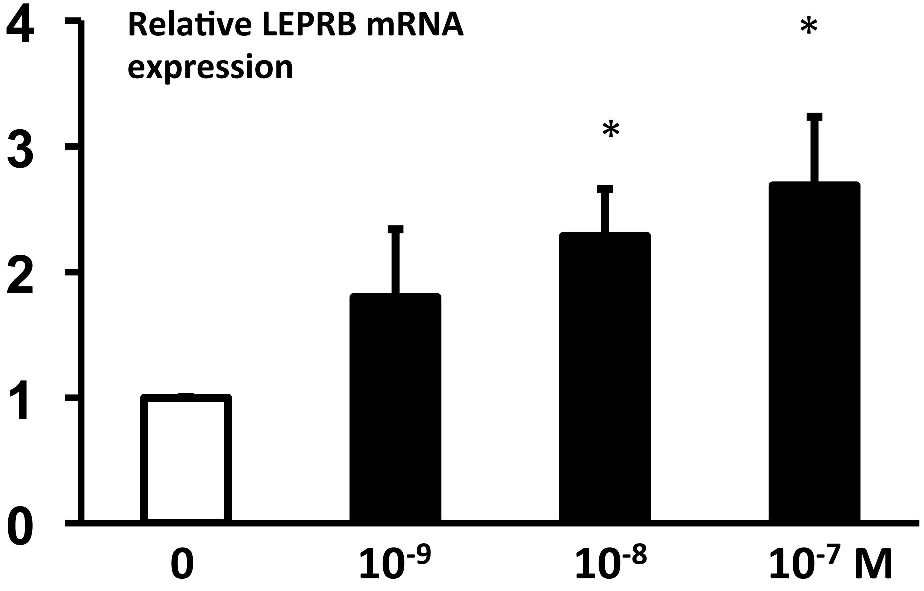

Suppl. Fig. 1-3

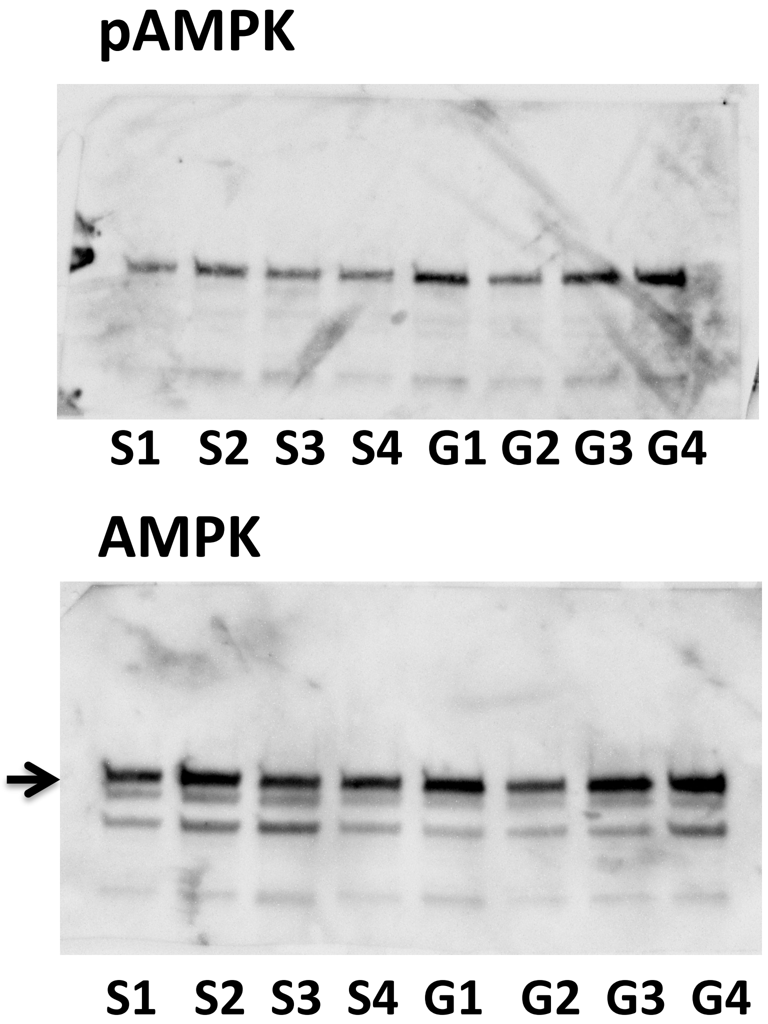

Suppl. Fig. 2-1

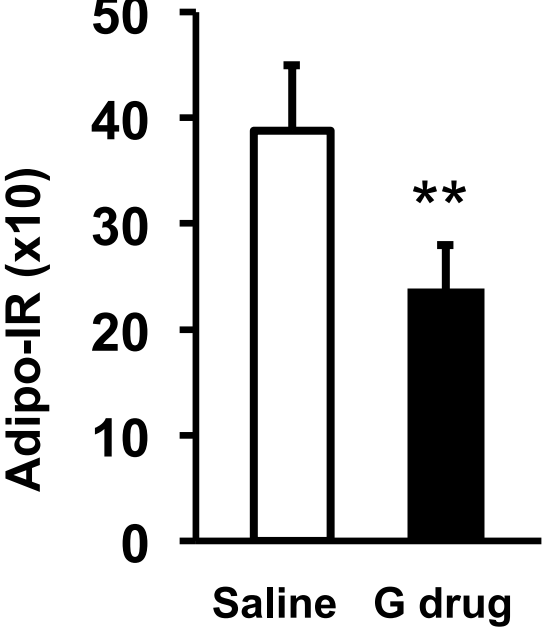

Suppl. Fig. 2-2

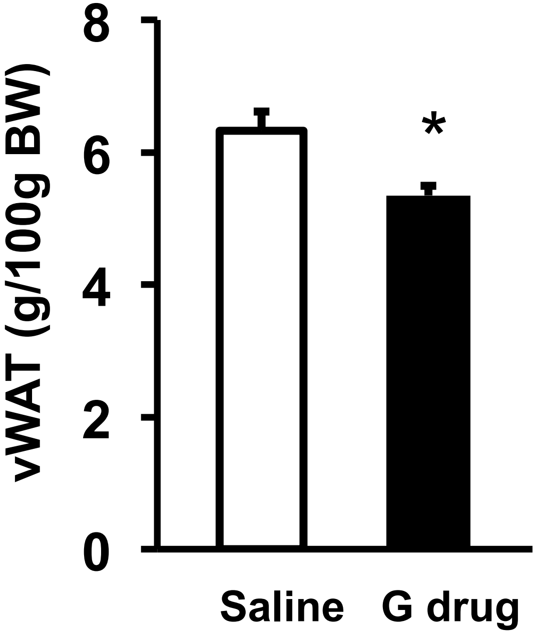

Suppl. Fig. 2-3

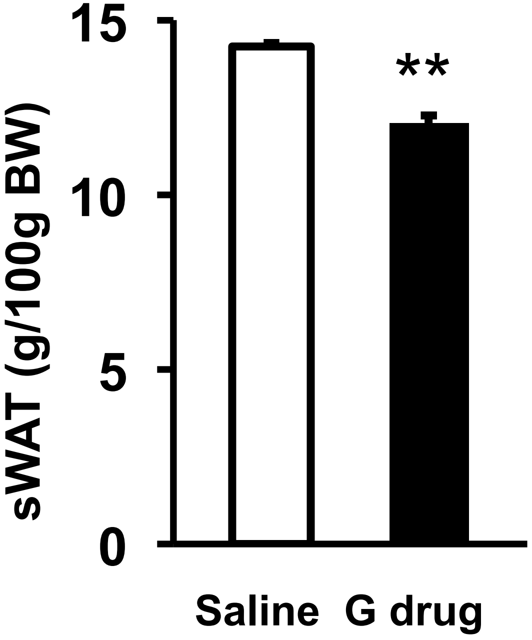

Suppl. Fig. 2-4

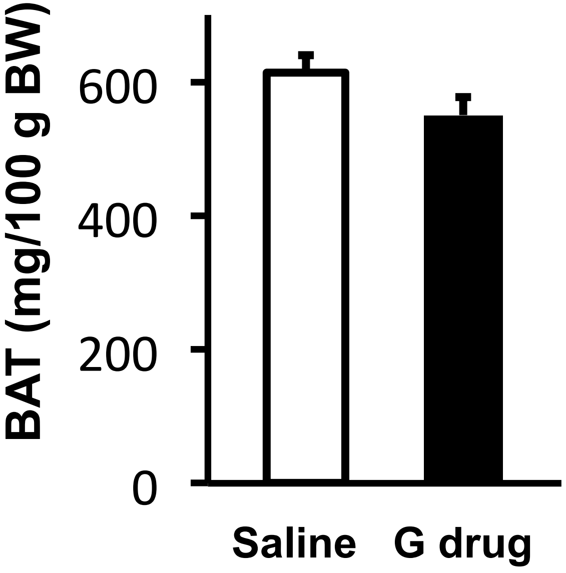

Suppl. Fig. 2-5

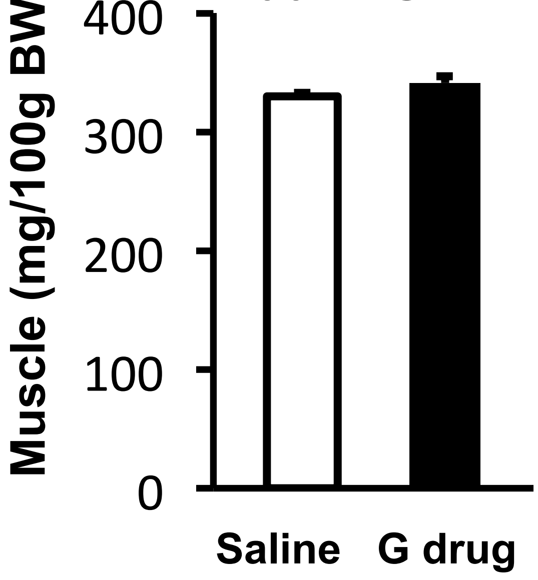

Suppl. Fig. 2-6

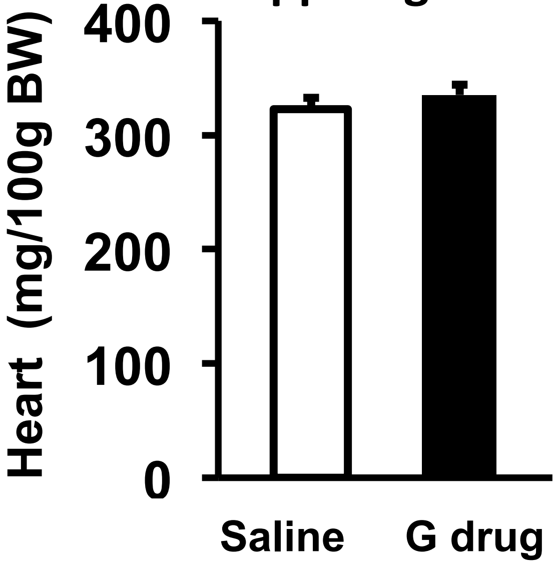

Suppl. Fig. 3-1

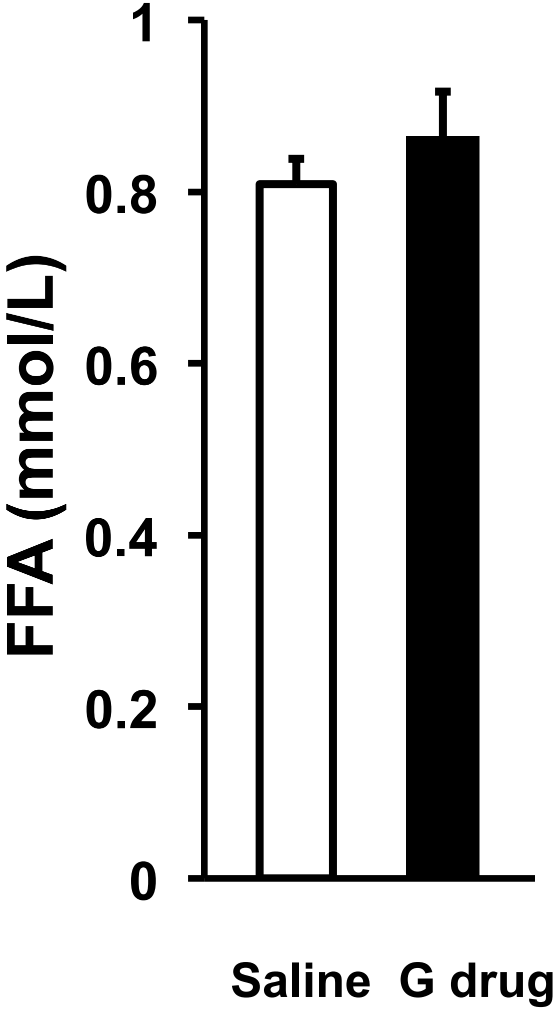

Suppl. Fig. 3-2

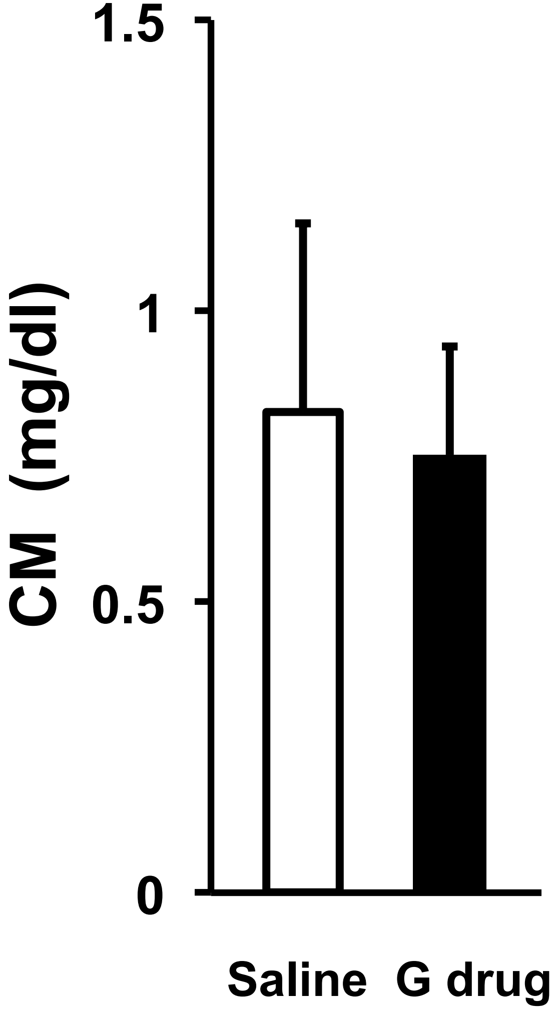

Suppl. Fig. 3-3

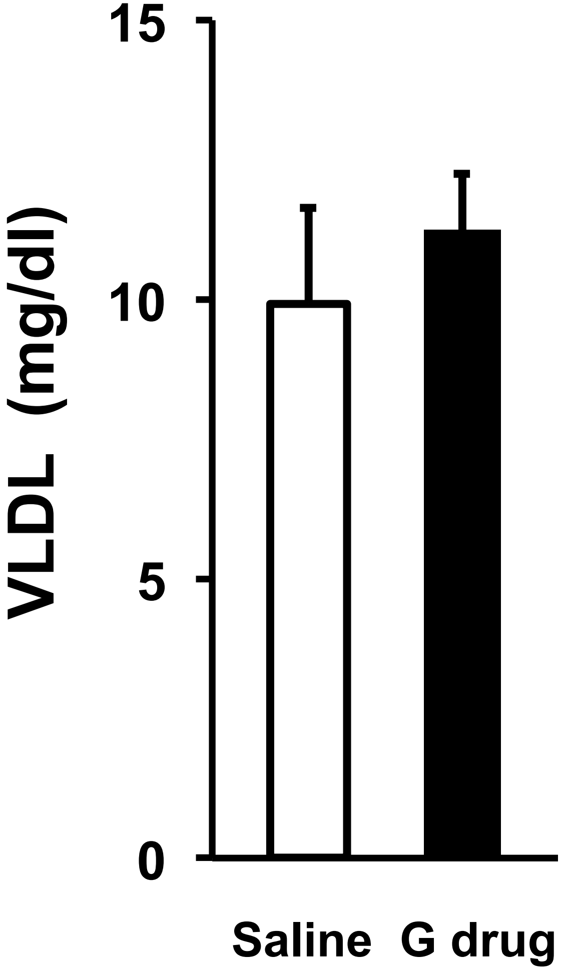

Supplement: Supplementary file 2 — Supplementary Figures [file 41598_2020_70689_MOESM2_ESM.pdf]
