# Supplementary material for: Administration of small-molecule guanabenz acetate attenuates fatty liver and hyperglycemia associated with obesity
Source: Sci Rep. 2020 Aug 13;10:13671. doi: 10.1038/s41598-020-70689-5 (PMC7426972; doi:10.1038/s41598-020-70689-5)
Supplement: Supplementary file 3 — Supplementary Table [file 41598_2020_70689_MOESM3_ESM.pdf]

Administration of small-molecule guanabenz acetate attenuates fatty liver and hyperglycemia associated with obesity

Satoshi Yoshino, Yusaku Iwasaki, Shunichi Matsumoto, Tetsurou Satoh, Atsushi Ozawa, Eijiro Yamada, Satoru Kakizaki, Juan Alejandro Oliva Trejo, Yasuo Uchiyama, Masanobu Yamada and Masatomo Mori

Table 1. Abbreviations.

Ab, antibody
Adipo-IR, adipose tissue insulin resistance
AMPK, AMP-activated protein kinase
BAT, brown adipose tissue
BODIPY, boron-dipyrromethene
BW, body weight
CHREBP, charbohydrate responsive element binding protein
CM, chylomicron
CPT, carnitine palmytoil transferase
ELISA, enzyme-linked immuno sorbent assay
FA, fatty acid
Fig. Figure
FFA, free fatty acid
GLP-1, glucagon-like peptide-1
GPAT, glycerol 3-phosphate acyltransferase
G3PD, glyceraldehyde-3-phosphate dehydrogenase
Helz2, Helicase with zinc finger 2
HFD, high-fat diet
HOMA, homeostasis model assessment
HOMA-IR, HOMA of insulin resistance
HOMAß, HOMA ß-cell function
LCAD, long-chain acyl-CoA dehydrogenase
Leprb, Leptin receptor long form
LFD, low-fat diet
MGAT, monoacylglycerol-O-acyltransferase
MTP, microsomal triglyceride transfer protein
NASH, nonalcoholic steatohepatitis
PPAR, peroxisome proliferator-activated receptor
PGC, PPAR-γ coactivator
PDIP, PPAR-γ-DNA-binding domain-interacting protein
qRT-PCR, quantitative real time polymerase chain reaction
SCD, stearoyl-CoA desaturase
SREBP, sterol regulatory element-binding protein

sWAT , subcutaneous WAT
Suppl. Supplementary
T2DM, type2 diabetes mellitus
UCP, uncoupling protein
vWAT, visceral WAT,
VLDL, very low density lipoprotein
WAT, white adipose tissue
